# Reviewers

**DOI:** 10.1111/cas.16031

**Published:** 2023-12-18

The publication of invaluable papers in Cancer Science depends on the prompt, careful review of submitted manuscripts. We would like to thank the following experts for reviewing manuscripts submitted between October 1, 2022 to September 30, 2023.

Reviewer names

Hiroyuki Abe

Tatsuya Abe

Keishi Adachi

Masahiro Adachi

João Agostinho Machado‐Neto

Shusuke Akamatsu

Yoshiki Akatsuka

Hiroshi Akazawa

Yoshimitu Akiyama

Kiyohiro Ando

Koji Ando

Shigeki Aoki

Yoshiki Arakawa

Norie Araki

Hideyuki Arita

Ken Asada

Naoko Asano

Tetsuhiko Asao

Eishi Ashihara

Teijiro Aso

Eishi Baba

Hideo Baba

Yoshifumi Baba

Hiroko Bando

Yasuko Bando

Narikazu Boku

Maria J Brito

Horacio Cabral

Shang Cai

Zhixiong Cai

Marco Candeias

Ke Cao

Ylenia Capodanno

Amancio Carnero

Fabiola Attie De Castro

Stefania Catalano

Marcello Ceci

Lo‐Kong Chan

Chien‐Wen Chen

Xu Chen

Ying Chen

Yu Chen

Quan Cheng

Hideki Chiba

Natsuko Chiba

Shigeru Chiba

Tomoki Chiba

Yang Chi‐Chun

Kazuaki Chikamatsu

Carlos Takahiro Chone

Yu‐Ting Chou

Pedro Coelho

Lucy Cook

Jaroslaw Czyz

Thao Thi Dang

Saumitra Das

Xiaofan Ding

Takahiro Domoto

Nicolas Duployez

Takashi Ebihara

Nagayasu Egawa

Hidetoshi Eguchi

Shogo Ehata

Pieter Eichhorn

Takahiro Einama

Daisuke Ennishi

Hideki Enokida

Tomohiro Enokida

Bo Fan

Changfa Fan

Li Fu

Li‐Wu Fu

Shigeo Fuji

Hirofumi Fujii

Hodaka Fujii

Satoshi Fujii

Shin‐Ichiro Fujii

Kiyohide Fujimoto

Naohiro Fujimoto

Takao Fujisawa

Yasuhiro Fujisawa

Teruaki Fujishita

Kazutoshi Fujita

Toshitsugu Fujita

Yu Fujita

Hiroshi Fujiwara

Naoto Fujiwara

Yutaka Fujiwara

Akihisa Fukuda

Tomohiko Fukuda

Hiroshi Fukuhara

Takayuki Fukui

Kohei Fukuoka

Noriyasu Fukushima

Noriyoshi Fukushima

Satoshi Fukushima

Takuya Fukushima

Wakaba Fukushima

Itoh Fumiko

Tatsuhiko Furukawa

Jun‐Ichi Furuta

Nozomu Fuse

Mitsuru Futakuchi

Manabu Futamura

Mingwe Gao

Gemma Gatta

Morteza Gholami

Gary S Goldberg

Akiteru Goto

Atsushi Goto

Hiroaki Goto

Mariko Goto

Jih‐Hwa Guh

Ruixia Guo

Yoshimi Haga

Gordana Halec

Akinobu Hamada

Shin Hamada

Yasuo Hamamoto

Chisato Hamashima

Ichiro Hanamura

Toru Hanamura

Hiroshi Handa

Megumi Hara

Hironori Harada

Hiroshi Harada

Koji Haratani

Koichiro Haruki

Masayuki Haruta

Daigo Hashimoto

Itaru Hashimoto

Masakazu Hashimoto

Naoya Hashimoto

Shinichi Hashimoto

E Paul Hasty

Yutaka Hata

Hiroto Hatakeyama

Shigetsugu Hatakeyama

Mami Hatano

Yoshihiro Hatta

Naoko Hattori

Yutaka Hattori

Michael Hausmann

Fumihiko Hayakawa

Yoku Hayakawa

Hidetoshi Hayashi

Hideyuki Hayashi

Takuo Hayashi

Tsutomu Hayashi

Yoshihiro Hayashi

Eric Hervouet

Yasuhiro Hida

Nobuaki Higashi

Shinjiro Hino

Kunihiko Hinohara

Kenichi Hirabayashi

Toru Hiraga

Atsushi Hiraoka

Nobuyoshi Hiraoka

Akira Hirasawa

Eishu Hirata

Makoto Hirata

Sachie Hiratsuka

Yoshihiko Hirohashi

Shuichi Hironaka

Seiko Hirono

Yuichi Hirose

Shigeo Hisamori

Asahi Hishida

Takaki Hiwasa

Shohei Honda

Young Kwon Hong

Fumiya Hongo

Tsunaki Hongu

Yoshitaka Honma

Kanya Honoki

Akira Horii

Rie Horii

Yoshiya Horimoto

Hidehito Horinouchi

Keisuke Horiuchi

Naoki Hosen

Sodai Hoshiai

Ayuko Hoshino

Daisuke Hoshino

Naoko Hosono

Satoyo Hosono

Noriko Hosoya

Quingjiang Hu

Canhua Huang

Chensong Huang

David Hughes

Daisuke Ichikawa

Masashi Idogawa

Tomoko Iehara

Kazuhiko Igarashi

Taro Iguchi

Hisashi Iizasa

Hideaki Ijichi

Kazuhiro Ikeda

Sadakatsu Ikeda

Satoshi Ikeda

Tohru Ikeda

Tsuneo Ikenoue

Takayuki Ikezoe

Yusuke Imagawa

Toshio Imai

Yoichi Imai

Masamichi Imajo

Tatsuhiko Imaoka

Yoshimi Imawari

Kazuhiko Ino

Junichi Inokuchi

Akira Inoue

Katarzyna Inoue

Kenichi Inoue

Masahiro Inoue

Minoru Inoue

Yasumichi Inoue

Takashi Inozume

Masafumi Inui

Jun Ishihara

Junko Ishihara

Takaya Ishihara

Hiroki Ishii

Kenichiro Ishii

Takamichi Ishii

Eiichi Ishikawa

Hitoshi Ishikawa

Rei Ishikawa

Yuichi Ishikawa

Takatsugu Ishimoto

Toru Ishitani

Kenji Ishitsuka

Kenichi Ishizu

Kota Itahashi

Hidemi Ito

Ken‐Ichi Ito

Takeshi Ito

Yuri Ito

Shinji Itoh

Susumu Itoh

Atsushi Iwama

Eiji Iwama

Shintaro Iwama

Masaaki Iwatsuki

Naoki Izawa

Kouji Izumi

Koji Izutsu

Luis Jave‐Suárez

Krishnaraj Jayaraman

Keun‐Yeong Jeong

Yoshikazu Johmura

Tetsuya Kadonosono

Norimitsu Kadowaki

Shigenori Kadowaki

Hiroshi Kagamu

Hidenori Kage

Yuki Kagoya

Taisuke Kajino

Hiroaki Kajiyama

Anna Kakehashi

Yoshihiro Kakeji

Hideaki Kakeya

Nobuyuki Kakiuchi

Kazuharu Kamachi

Walied Kamel

Yasuhiko Kamikubo

Masashi Kanai

Takayuki Kanaseki

Hiroaki Kanda

Shuzo Kaneko

Dongchon Kang

Mitsunobu Kano

Michalis V Karamouzis

Takahiro Karasaki

Kennosuke Karube

Atsuko Kasahara

Hiroaki Kasashima

Katsumi Kasashima

Akiyoshi Kasuga

Chikatoshi Katada

Kota Katanoda

Hiroaki Kataoka

Hiromi Kataoka

Kazuhiro Katayama

Kikuya Kato

Naoya Kato

Shigeaki Kato

Shingo Kato

Taigo Kato

Takuma Kato

Takuya Kato

Yukinari Kato

Hiroto Katoh

Keisuke Katsushima

Tsuyoshi Kawabata

Manabu Kawada

Kosuke Kawaguchi

Takumi Kawaguchi

Masataka Kawai

Hisato Kawakami

Daisuke Kawakita

Yawara Kawano

Yoshihiro Kawasaki

Atsunari Kawashima

Akihito Kawazoe

Masahito Kawazu

Tanaka Kazuhiro

Hirotsugu Kenmotsu

Hiroyasu Kidoya

Toru Kiguchi

Eiji Kikuchi

Koji Kikuchi

Yoshikane Kikushige

Hiroshi Kimura

Keiji Kimura

Takahiro Kimura

Yukiko Kiniwa

Ichiro Kinoshita

Manabu Kinoshita

Seigo Kinuya

Hiroyuki Kishi

Hidenori Kitai

Hiroshi Kitajima

Shunsuke Kitajima

Hidemitsu Kitamura

Katsuyuki Kiura

Shinichi Kiyonari

Kazuma Kiyotani

Akira Kobayashi

Hiroki Kobayashi

Hiroshi Kobayashi

Hiroya Kobayashi

Miho Kobayashi

Shogo Kobayashi

Susumu Kobayashi

Takashi Kobayashi

Yoshihisa Kobayashi

Masayoshi Kobune

Yuta Kochi

Takahiro Kodama

Yuzo Kodama

Takahiro Kogawa

Yasunori Kogure

Kenichi Kohashi

Susumu Kohno

Shinji Kohsaka

Motohiro Kojima

Takashi Kojima

Yasushi Kojima

Yu‐Ichiro Koma

Masaaki Komatsu

Shuhei Komatsu

Yoshito Komatsu

Keigo Komine

Daisuke Komura

Kazumasa Komura

Shunsuke Kon

Eisaku Kondo

Eisei Kondo

Takeshi Kondo

Akimitsu Konishi

Hirotaka Konishi

Masamitsu Konno

Keiko Kono

Tsuyoshi Konuma

Chihaya Koriyama

Takeo Kosaka

Naohiko Koshikawa

Ai Kotani

Hiroshi Kotani

Takenori Kotani

Nobuji Kouno

Junji Koya

Shohei Koyama

Yuriko Koyanagi

Terufumi Kubo

Yoshiaki Kubota

Takahiro Kuchimaru

Yasusei Kudo

Yujin Kudo

Rei Kudor

Iwao Kukimoto

Shogo Kumagai

Makoto Kunisada

Yutaka Kurebayashi

Kozo Kuribayashi

Sei Kuriyama

Junya Kuroda

Masahiko Kuroda

Daisuke Kurotaki

Kazuhiko Kurozumi

Masahiko Kusumoto

Shigeru Kusumoto

Takeshi Kuwata

Jung Weon Lee

Yuehchun Lee

Anton Lennikov

Chia‐Wei Li

Chia‐Yang Li

Jian‐Ming Li

Lianbo Li

Wentong Li

Xiao‐Nan Li

Yandong Li

Yanjing Li

Yu Li

Yingsong Lin

Qiang Liu

Shihai Liu

Tao Liu

Thanpisit Lomphithak

Siew‐Kee Low

Jun‐Hang Luo

Ming Luo

Xiao‐Bin Lv

Yanlei Ma

Mitsuhiro Machitani

Daichi Maeda

Osamu Maeda

Takahiro Maeda

Yoshinobu Maeda

Tomohiko Maehama

Makoto Maemondo

Hideki Makinoshima

Hideki Makishima

Shinichi Makita

Lamjed Mansour

Constantino Martinez

Yoshiaki Maru

Dai Maruyama

Reo Maruyama

Yasufumi Masaki

Yosuke Masamoto

Atsushi Masamune

Tetsuo Mashima

Kenta Masuda

Kiyoshi Masuda

Mari Masuda

Shinobu Masuda

Takaaki Masuda

Yohei Masugi

Kenta Masui

Daisuke Matsubara

Satoru Matsuda

Tomohiro Matsuda

Hirotaka Matsui

Akinobu Matsumoto

Kazuhiro Matsumoto

Shinji Matsumoto

Tomonori Matsumoto

Yoshihisa Matsumoto

Noriomi Matsumura

Yoshihiro Matsumura

Keisuke Matsusaka

Kazuyuki Matsushita

Juntaro Matsuzaki

Shuji Mikami

Kosuke Mima

Takashi Minami

Yosuke Minami

Yuko Minami

Sachiko Minamiguchi

Yoji Minamishima

Kenji Mishima

Saori Mishima

Yuichi Mitobe

Shuichi Mitsunaga

Masanao Miwa

Etsuko Miyagi

Yohei Miyagi

Hideaki Miyake

Makito Miyake

Toshihiro Miyamoto

Tsutomu Miyamoto

Akihiko Miyanaga

Shin‐Ichi Miyatake

Eisaku Miyauchi

Kohta Miyawaki

Hiroyuki Miyoshi

Yasuo Miyoshi

Masahiro Mizoguchi

Hideaki Mizuno

Kazunori Mizuno

Ryuichi Mizuno

Satsuki Mochizuki

Isao Momose

Shuji Momose

Yukihide Momozawa

Keiichiro Mori

Seiichi Mori

Yuji Morine

Masahiro Morise

Kazuhiro Morishita

Ken Morita

Chigusa Morizane

Toshiro Moroishi

Matteo Morotti

Noriko Motoi

Kazuya Motomura

Yashiro Motooka

Noboru Motoyama

Hidefumi Mukai

Naofumi Mukaida

Ken‐Ichi Mukaisho

Toru Mukohara

Wataru Munakata

Jordi Muntane

Kazuhiro Murakami

Kosaku Murakami

Kosuke Murakami

Naoya Murakami

Takashi Murakami

Yoshiki Murakumo

Daisuke Muraoka

Takayuki Murata

Naoko Murata‐Kamiya

Masafumi Muratani

Takahiko Murayama

Manabu Muto

Satoshi Muto

Michihiro Mutoh

Richard Naftalin

Genta Nagae

Hirokazu Nagai

Motoo Nagane

Hiroaki Nagano

Osamu Nagano

Toshitaka Nagao

Koji Nagaoka

Kazunori Nagasaka

Shun Nagashima

Yoshiaki Nagatani

Satoshi Nagayama

Yoichi Naito

Yuji Naito

Yutaka Naito

Kazuhito Naka

Hayato Nakagawa

Masao Nakagawa

Shigeki Nakagawa

Takuya Nakagawa

Tohru Nakagawa

Tomomi Nakahara

Yousuke Nakai

Hideaki Nakajima

Yu‐Ichiro Nakajima

Katsumasa Nakamura

Kenichi Nakamura

Motoki Nakamura

Naoya Nakamura

Ritsuko Nakamura

Seigo Nakamura

Takashi Nakamura

Yoshiaki Nakamura

Yuki Nakanishi

Hiroyasu Nakano

Hisashi Nakano

Takashi Nakaoku

Chiaki Nakaseko

Ayako Nakashoji

Kayo Nakata

Mizuho Nakayama

Tomio Nakayama

Yozo Nakazawa

Takeshi Namekawa

Takeshi Namiki

Yasuhito Nannya

Atsushi Nara

Shintaro Narita

Yoshitaka Narita

Ken Natsuga

Manabu Natsumeda

Seiji Niho

Jun Nishida

Mikako Nishida

Tetsuya Nishida

Tadaaki Nishikawa

Yuji Nishikawa

Momoko Nishikori

Tatsunori Nishimura

Hiroshi Nishina

Takashi Nishina

Tomohiro Nishina

Hitomi Nishinakamura

Yoshikazu Nishino

Makoto Nishio

Shin Nishio

Kentaro Nishioka

Yasuhiko Nishioka

Michiru Nishita

Hideki Nishitoh

Kohji Noguchi

Rei Noguchi

Takuro Noguchi

Satoshi Nojima

Kisato Nosaka

Atsushi Oba

Makoto Oba

Shino Oba

Kazutaka Obama

Yuki Obata

Mieko Ochi

Sadahisa Ogasawara

Mikako Ogawa

Hirokazu Ogino

Hisakazu Ogita

Takashi Ohama

Akihiro Ohashi

Kadoaki Ohashi

Shigeo Ohba

Hiroto Ohguchi

Tatsuo Ohira

Tomokazu Ohishi

Fumiharu Ohka

Takayuki Ohkuri

Akihiro Ohmoto

Makoto Ohno

Shinobu Ohnuma

Nobumichi Ohoka

Kenji Ohshima

Tomohiko Ohta

Takao Ohtsuka

Yoshihiro Ohue

Naoki Oishi

Satoshi Oizumi

Yusuke Oji

Hidenori Ojima

Atsushi Okabe

Nobuyuki Okahashi

Wataru Okamoto

Eiji Oki

Hajime Okita

Yukari Okita

Mistuaki Okodo

Shujiro Okuda

Yoshinaga Okugawa

Yusuke Okuma

Shintaro Okumura

Chitose Oneyama

Rikiya Onimaru

Nobuyuki Onishi

Atsushi Ono

Hiroaki Ono

Makiko Ono

Mayumi Ono

Yasuhito Onodera

Kunishige Onuma

Akira Orimo

Mitsuhiko Osaki

Takahiro Osawa

Tsuyoshi Osawa

Hiroko Oshima

Atsushi Osoegawa

Motoyuki Otsuka

Takemi Otsuki

Kota Ouchi

Yuko Oya

Toshinori Ozaki

Yukinori Ozaki

Yuki Ozato

Yuichi Ozawa

Isao Oze

Elisabetta Palazzo

Cheng Pan

Amanda Panfil

Jae‐Hyun Park

Shiqian Qi

Susumu Rokudai

Katarzyna Rygiel

Hiroshi Saeki

Sumit Sahni

Akira Saito

Ayumi Saito

Takuro Saito

Tatsuya Saito

Masao Saitoh

Tomohiko Sakabe

Kimiyoshi Sakaguchi

Akira Sakai

Kazuko Sakai

Naoya Sakamoto

Takeharu Sakamoto

Tomohiro Sakamoto

Yoshihiro Sakamoto

Shingo Sakashita

Tomohisa Sakaue

Yasunaru Sakuma

Yuji Sakuma

Hideyuki Sakurai

Yu Sakurai

Oltea Sampetrean

Masashi Sanada

Koji Sasaki

So‐Ichiro Sasaki

Megumi Sasatani

Goro Sashida

Akira Sato

Mitsuo Sato

Yasushi Sato

Yusuke Sato

Felix Schmitt

Jurian Schuijers

Masahiro Seike

Ikuo Sekine

Shigeki Sekine

Xingjuan Shi

Aya Shiba

Satoshi Shiba

Atsushi Shibata

Norihito Shibata

Kazuhiko Shien

Kohei Shigeta

Po‐Chang Shih

Sho Shiino

Kazuyuki Shimada

Mitsuo Shimada

Shu Shimada

Teppei Shimamura

Hideyuki Shimizu

Junichi Shimizu

Takatsune Shimizu

Kazuya Shimoda

Masayuki Shimoda

Tatsunori Shimoi

Tomoya Shimokata

Keiko Shinjo

Naoki Shinojima

Yasushi Shintani

Daisuke Shiokawa

Masaki Shiota

Bunsyo Shiotani

Atsushi Shiozaki

Kouya Shiraishi

Masayuki Shirasawa

Hirokazu Shoji

Iwona Sidorkiewicz

Siro Simizu

Eric Snyder

Takanori So

Yasushi Soda

Kenzo Soejima

Kenbun Sone

Yukihiko Sonoda

Takashi Sonoki

Masahiro Sonoshita

Norbert Stefan

Goki Suda

Kenichi Suda

Yoshiyuki Suehara

Kokichi Sugano

Keishi Sugimachi

Kenji Sugio

Reiko Sugiura

Tsuyoshi Sugiura

Daisuke Sugiyama

Hiromi Sugiyama

Hidetoshi Sumimoto

Gongping Sun

Masaki Sunagawa

Yu Sunakawa

Kuniko Sunami

Ayako Suzuki

Hidefumi Suzuki

Hiromichi Suzuki

Hiroshi I Suzuki

Hiroyuki Suzuki

Koji Suzuki

Miho Suzuki

Mikiko Suzuki

Rei Suzuki

Shugo Suzuki

Takafumi Suzuki

Takeshi Suzuki

Toshihiro Suzuki

Junji Suzumiya

Takahiro Tabuchi

Hiroshi Tada

Masatoshi Tagawa

Ayumi Taguchi

Ayumu Taguchi

Keiko Taguchi

Mamoru Takada

Masatoshi Takagi

Satoshi Takagi

Akiko Takahashi

Hirotaka Takahashi

Kei Takahashi

Masanobu Takahashi

Masayuki Takahashi

Ryou‐U Takahashi

Tsuyoshi Takahashi

Yoshinori Takahashi

Tomoiku Takaku

Manabu Takamatsu

Hirokazu Takami

Shinkichi Takamori

Kiyoko Takane

Ken Takasawa

Hiroki Takashima

Atsushi Takatori

Kenichi Takayama

Koichi Takayama

Haruhiko Takeda

Haruna Takeda

Kazuyoshi Takeda

Masayuki Takeda

Tetsuo Takehara

Hisanori Takenobu

Hideyuki Takeshima

Fumitaka Takeshita

Shinji Takeuchi

Yasuto Takeuchi

Toshiro Takezaki

Tetsuro Taki

Nagio Takigawa

Yuichi Takiguchi

Masahiro Takikawa

Takahisa Takino

Jun Takizawa

Keisuke Tamari

Kazuo Tamura

Takaaki Tamura

Yasuaki Tamura

Masahiko Tanabe

Fumiaki Tanaka

Fumihiro Tanaka

Hajime Tanaka

Ichidai Tanaka

Kousuke Tanaka

Miwa Tanaka

Nobuyuki Tanaka

Shota Tanaka

Yasuhito Tanaka

Yosuke Tanaka

Masahiro Tanemura

Hailin Tang

Jian Tang

Masaji Tani

Hiroaki Taniguchi

Hiroya Taniguchi

Kohei Taniguchi

Koji Taniguchi

Chizu Tanikawa

Takashi Tanikawa

Akihide Tanimoto

Keiji Tanimoto

Yukari Taniyama

Yongguang Tao

Woan‐Yuh Tarn

Etsu Tashiro

Satoshi Tashiro

Masashiro Tatebe

Keisuke Tateishi

Kensuke Tateishi

Ryosuke Tateishi

Hiro Tatetsu

Yasutoshi Tatsumi

Kenji Tatsuno

Hiroshi Tazawa

Naoki Terada

Koji Teramoto

Toshiki Terao

Keita Terashima

Takeshi Terashima

Yasuhito Terui

Martin K Thomsen

Hua Tian

Suyan Tian

Emi Tokuda

Eriko Tokunaga

Fuminori Tokunaga

Shuta Tomida

Yoshito Tomimaru

Akihiro Tomita

Shuhei Tomita

Arata Tomiyama

Satoshi Tomizawa

Takashi Toya

Tatsuya Toyama

Hidenori Toyoda

Gouji Toyokawa

Julia Tsang

Ayumu Tsubosaka

Shoma Tsubota

Kunihiro Tsuchida

Kenji Tsuchihashi

Hiroyuki Tsuchiya

Kosuke Tsuchiya

Naoto Tsuchiya

Hiroyuki Tsuda

Masumi Tsuda

Akihito Tsuji

Takemasa Tsuji

Takahiro Tsujikawa

Tohru Tsujimura

Tomohide Tsukahara

Hirotake Tsukamoto

Tetsuya Tsukamoto

Kunihiro Tsukasaki

Yoko Tsukita

Tadasuke Tsukiyama

Tatsuhiko Tsunoda

Koichiro Tsutsumi

Toyonori Tsuzuki

Shizuka Uchida

Takeshi Uchiumi

Masashi Ueda

Yoshihide Ueda

Yutaka Ueda

Wataru Uegami

Satoshi Ueha

Hisanori Uehara

Hiroji Uemura

Motohide Uemura

Sayaka Ueno

Takayuki Ueno

Tomotaka Ugai

Atsushi Umemura

Shigeki Umemura

Fumihiko Urabe

Milena Urbini

Hiroshi Ureshino

Wataru Usuba

Yoshiaki Usui

Kensuke Usuki

Katsuhiro Uzawa

Sergi Vidal‐Sicart

Dominic Voon

Keiko Wada

Satoshi Wada

Hiroaki Wakimoto

Daiko Wakita

Jing H H Wang

Jinxi Wang

Min Wang

Tadashi Watabe

Keisuke Watanabe

Kenji Watanabe

Kousuke Watanabe

Nobumoto Watanabe

Satoshi Watanabe

Yukihide Watanabe

Haiming Wei

Liang Weng

Seo Wooseok

Fu‐Zong Wu

Shunbin Xiong

Naihan Xu

Norikazu Yabuta

Hiroshi Yagi

Koichi Yagi

Shigeki Yagyu

Hideaki Yahata

Daisaku Yamada

Tadaaki Yamada

Takeshi Yamada

Tesshi Yamada

Yuma Yamada

Makoto Yamagishi

Hideki Yamaguchi

Hiroki Yamaguchi

Kiyoshi Yamaguchi

Motoko Yamaguchi

Noritaka Yamaguchi

Rui Yamaguchi

Yoshifumi Yamaguchi

Taiki Yamaji

Hayashi Yamamoto

Kazuhito Yamamoto

Mizuki Yamamoto

Noboru Yamamoto

Seiichiro Yamamoto

Yuki Yamamoto

Yusuke Yamamoto

Yoshihisa Yamano

Kazuhiro Yamanoi

Osamu Yamasaki

Takahiro Yamasaki

Keishi Yamashita

Kyoko Yamashita

Satoshi Yamashita

Taro Yamashita

Tatsuya Yamashita

Toshinari Yamashita

Takahiro Yamauchi

Etsuko Yamazaki

Jianyun Yan

Noriko Yanagitani

Izumi Yanatori

Minglei Yang

Tao Yang

Yeran Yang

Yinlong Yang

Hiromu Yano

Jun Yao

Yunjin Yao

Hiroyuki Yasuda

Jun Yasuda

Takahiko Yasuda

Koya Yasukawa

Masahiro Yasunaga

Akitada Yogo

Takehiko Yokobori

Akira Yokoi

Akihiko Yokoyama

Akira Yokoyama

Satoru Yokoyama

Yasuhisa Yokoyama

Hiroshi Yokozaki

Kimio Yonesaka

Wu Yongbing

Keisuke Yoshida

Kenichi Yoshida

Kosuke Yoshida

Tatsuya Yoshida

Kosuke Yoshihara

Yasuhiro Yoshimatsu

Akihide Yoshimi

Yuya Yoshimoto

Kiyoshi Yoshimura

Michio Yoshimura

Hiromasa Yoshioka

Kenichi Yoshioka

Yasuo Yoshioka

Yusuke Yoshioka

Tadamasa Yoshitake

Tomokazu Yoshizaki

Akihiko Yoshizawa

Shi‐Tong Yu

Shyng‐Shiou Yuan

Yoshitaka Zenke

Rihong Zhai

Weiyu Zhang

Yongliang Zhang

Yu Zhang

Yuchen Zhang

Chengquan Zhao

Peiqing Zhao

Yang Zhao

Chao Zheng

Ling Zheng

Lai‐Ping Zhong

Jingying Zhou

Guangsheng Zhu

Guiquan Zhu

Ting Zhuang

